# Antimicrobial Resistance in *Escherichia coli* Strains Isolated from Humans and Pet Animals

**DOI:** 10.3390/antibiotics10010069

**Published:** 2021-01-13

**Authors:** Nikola Puvača, Rosa de Llanos Frutos

**Affiliations:** 1 Faculty of Biomedical and Health Sciences, Jaume I University, Avinguda de Vicent Sos Baynat, s/n, 12071 Castelló de la Plana, Spain; al409850@uji.es; 2 Department of Engineering Management in Biotechnology, Faculty of Economics and Engineering Management in Novi Sad, University Business Academy in Novi Sad, Cvećarska 2, 21000 Novi Sad, Serbia

**Keywords:** antimicrobial resistance, antibiotics, public health, microbiology, *E. coli*

## Abstract

Throughout scientific literature, we can find evidence that antimicrobial resistance has become a big problem in the recent years on a global scale. Public healthcare systems all over the world are faced with a great challenge in this respect. Obviously, there are many bacteria that can cause infections in humans and animals alike, but somehow it seems that the greatest threat nowadays comes from the *Enterobacteriaceae* members, especially *Escherichia coli*. Namely, we are witnesses to the fact that the systems that these bacteria developed to fight off antibiotics are the strongest and most diverse in *Enterobacteriaceae*. Our great advantage is in understanding the systems that bacteria developed to fight off antibiotics, so these can help us understand the connection between these microorganisms and the occurrence of antibiotic-resistance both in humans and their pets. Furthermore, unfavorable conditions related to the ease of *E. coli* transmission via the fecal–oral route among humans, environmental sources, and animals only add to the problem. For all the above stated reasons, it is evident that the epidemiology of *E. coli* strains and resistance mechanisms they have developed over time are extremely significant topics and all scientific findings in this area will be of vital importance in the fight against infections caused by these bacteria.

## 1. Introduction

Scientists all over the world have studied *Escherichia coli* and it appears to be the most thoroughly investigated and best understood of all model microorganisms [1,2,3,4]. We already know that it is one of the first bacteria that colonizes the human gut immediately after birth [5,6,7]. On the other hand, *E. coli* is often the main culprit of infections in the gastrointestinal tract [8], as well as other parts of human and animal organisms [9,10]. In more precise terms, *E. coli* typically causes urinary infections [11,12], but it can also lead to many other serious infections and conditions, such as: appendicitis [13], pneumonia [14], meningitis [15], endocarditis [16], gastrointestinal infections [17], etc. Research findings have shown us that *E. coli* can cause infections in all age groups and those infections can be acquired in the general population, i.e., community-acquired, as well as related to healthcare institutions [18,19,20].

After Alexander Fleming had discovered penicillin in 1928, the whole course of medicine changed [21,22]. The revolutionary discovery of antibiotics made it possible for doctors to treat extremely severe cases of infectious diseases, which had previously been a very common cause of death [23,24]. That completely changed after antibiotics had been introduced and soon penicillin became the most widely used antibiotic in the world, saving millions of lives [25,26,27]. 

Unfortunately, only several years after doctors started using it in hospitals, the first cases of penicillin resistance by *Staphylococcus aureus* were identified [28]. Obviously, bacteria have managed to develop a system that can protect them and make them resistant to antibiotics [29]. Sadly, the situation with bacteria evolving resistance is getting worse day by day and we have literally come to a point when we can speak of the antimicrobial resistance presenting a worldwide problem [30,31,32,33,34,35,36].

When we speak about *E. coli*, the fact that it has been put on the World Health Organization’s (WHO) list that contains 12 families of bacteria that present the biggest danger to human health [37,38]. Ever since the first reported cases, *E. coli’s* resistance to antibiotic treatment has been continuously growing [39,40,41,42].

Scientific literature offers an abundance of research studies into the nature and behavior of *E. coli* [43,44,45,46]. The results point to several extremely interesting facts. This bacterium undoubtedly has considerable influence on human and animal lives [47,48], for the simple reason that it lives inside the gut and can very easily spread from fecal matter to the mouth [49,50]. Being the commensal bacteria of human and animal gut, it happens to be in close contact with numerous other bacteria [51]. However, perhaps the most fascinating thing about *E. coli* is its ability to pass on its genetic-resistant traits to microorganisms who share the same living environment, as well as to acquire resistance genes from them [52,53,54].

According to Poirel et al. [52] *E. coli* present a bacterium with a special place in the microbiological world since it can cause severe infections in humans and animals, and on the other hand represents a significant part of the autochthonous microbiota of the various hosts. The main apprehension is a transmission of virulent and resistant *E. coli* among animals and humans through various pathways. *E. coli* is a most important reservoir of resistance genes that may be accountable for treatment failures in both human and veterinary medicine [52]. An increasing number of resistance genes has been identified in *E. coli* isolates in the past 10 years, and many of these resistance genes were acquired by horizontal gene transfer. In the enterobacterial gene pool, *E. coli* acts as a donor and as a recipient of resistance genes and thereby can acquire resistance genes from other bacteria but can also pass on its resistance genes to other bacteria. Antimicrobial resistance in *E. coli* is considered one of the foremost disputes in both humans and animals at a global scale and needs to be considered as a real public health concern.

Barrios-Villa et al. [55] have observed increased evidence demonstrating the association between Crohn’s Disease (CD), a type of Inflammatory Bowel Disease (IBD), and non-diarrheagenic Adherent/Invasive *E. coli* (AIEC) isolates. Genomes of five AIEC strains isolated from individuals without IBD were sequenced and compared with AIEC prototype strains (LF82 and NRG857c), and with extra-intestinal uropathogenic strain (UPEC CFT073). Non-IBD-AIEC strains showed an Average Nucleotide Identity up to 98% compared with control strains. Blast identities of the five non-IBD-AIEC strains were higher when compared to AIEC and UPEC reference strains than with another *E. coli* pathotypes, suggesting a relationship between them [55]. In the same study, Barrios-Villa et al. [55], an incomplete Type VI secretion system was found in non-IBD-AIEC strains; however, the Type II secretion system was complete. Several groups of genes reported in AIEC strains were searched in the five non-IBD-AIEC strains, and the presence of *fimA*, *fliC*, *fuhD*, *chuA*, *irp2*, and *cvaC* were confirmed. Other virulence factors were detected in non-IBD-AIEC strains, which were absent in AIEC reference strains, including EhaG, non-fimbrial adhesin 1, PapG, F17D-G, YehA/D, FeuC, IucD, CbtA, VgrG-1, Cnf1, and HlyE. Based on the differences in virulence determinants and single-nucleotide polymorphisms (SNPs), it is plausible to suggest that non-IBD AIEC strains belong to a different pathotype.

Meanwhile, genomic analysis of *E. coli* strains isolated from diseased chicken in the Czech Republic [56] showed that multiresistant phenotype was detected in most of the sequenced strains with the predominant resistance to β-lactams and quinolones being associated with TEM-type beta-lactamase genes and chromosomal *gyrA* mutations. The phylogenetic analysis proved a huge variety of isolates that were derived from all groups. Clusters of closely related isolates within ST23 and ST429 indicated a possible local spread of these clones. Moreover, the ST429 cluster carried *bla*_CMY-2,− 59_ genes for AmpC β-lactamase and isolates of both clusters were well-equipped with virulence-associated genes, with significant variations in allocation of specific virulence-associated genes among phylogenetically distant lineages. Zoonotic APEC STs were also identified, such as ST117, ST354, and ST95, showing numerous molecular elements typical for human ExPEC [56]. 

As already stated, antibiotic resistance found in microorganisms presents a big challenge for medical practice in the whole world [57,58,59,60,61]. This is to a great extent the consequence of wrong or uncritical consumption of antibiotics. 

In a study by Abdelhalim et al. [62], from 17 Crohn’s disease patients and 14 healthy controls *E. coli* strains were isolated, 59% and 50% of them were identified as AIEC strains. It was discovered that *chuA* and *ratA* genes were the most significant genetic markers associated with AIEC compared to non-AIEC strains isolated from Crohn’s disease patients and healthy controls *p* = 0.0119, 0.0094, respectively. Most *E. coli* strains obtained from Crohn’s disease patients showed antibiotic resistance (71%) compared to healthy controls (29%) against at least one antibiotic. Investigation have demonstrated significant differences between AIEC strains and non-AIEC strains in terms of the prevalence of *chuA* and *ratA* virulence genes and the antibiotic resistance profiles. Furthermore, AIEC strains isolated from Crohn’s disease patients were found to be more resistant to β-lactam and aminoglycoside antibiotics than AIEC strains isolated from healthy controls [62].

*E. coli* strains isolated from animals in Tunisia [63] revealed occurrence of plasmid-mediated quinolone resistance between themselves. With 51 nalidixic acid-resistant isolates, 9 PMQR genes were harbored (5 co-harbored *qnrS1* and *qnrB1*, 3 harbored *qnrS1* and 1 harbored *qnrB1*). Two types of mutation in the QRDR of GyrA were observed: S83L and D87N. For the QRDR of ParC, the substitution S80I was observed as well, while A class 1 integron was found in isolates, respectively. The *tetA* or *tetB* gene was observed and both were co-harbored by two isolates. The *sul1*, *sul2*, and *sul3* genes were discovered, respectively. According to the presence of specific virulence genes, the nine strains were classified as UPEC, EAEC, and EPEC [63]. All mentioned highlight the plausible role of the avian industry as a reservoir of human pathogenic *E. coli* strains.

Yu et al. [64] have investigated the prevalence and antimicrobial-resistance phenotypes and genotypes of *E. coli* isolated from raw milk samples from mastitis cases in four regions of China. A total of 83 strains of *E. coli* were isolated and identified, but without any significant differences in the number of *E. coli* isolates detected among the two sampling seasons in the same regions. Nevertheless, a significant difference in *E. coli* prevalence was found among the four different regions. The isolates were most frequently resistant to penicillin (100%), acetylspiramycin (100%), lincomycin (98.8%), oxacillin (98.8%), and sulphamethoxazole (53%). All the *E. coli* strains were multiresistant to three antimicrobial classes, and the most frequent multidrug-resistance patterns for the isolates were resistant to three or four classes of drugs simultaneously [64].

In Egypt, Farhat et al. [65] have investigated the antimicrobial resistance patterns, the distribution of phylogenetic groups, and the prevalence and characteristics of integron-bearing *E. coli* isolates from outpatients with community-acquired urinary tract infections. A total of 134 human urine samples were positive for *E. coli*, from which a total of 80 samples were selected for further analyses. Most of the isolates (62.5%) proved multidrug resistance profiles. Group B2 was the most predominant phylogenetic group (52.5%), followed by group F (21.25%), Clade I or II (12.5%), and finally isolates of unknown phylogroup (13.75%). Of the 80 selected isolates, 7 of them carried class 1 integrons, which contained 3 different types of integrated gene cassettes, conferring resistance to streptomycin, trimethoprim, and some open reading frames of unknown function [65].

Low hygiene levels, lack of clean water, or poor sanitary conditions can create perfect conditions for the development and transmission of infections [66]. In addition to that, Farhani et al. [67] have total of 80 *E. coli* isolates, separated into 51 different genotypes. Using the Multi Locus VNTR Analysis (MLVA) profiles, a minimum spanning tree (MST) algorithm showed two clonal complexes with 71 isolates and only 9 isolates were stayed out of clonal complexes in the form of a singleton. High genotypic diversity was seen among *E. coli* strains isolated from hospital wastewaters; however, many isolates showed a close genetic relationship. Authors have concluded that MLVA as a rapid, inexpensive, and useful tool could be used for analysis of the phylogenetic relationships between *E. coli* strains [67].

Extended-spectrum beta-lactamases (ESBLs) are specific enzymes, which show resistance to almost all beta-lactam antibiotics [68], including penicillin [69], cephalosporin [70], etc. [71]. Cases of infections in which ESBLs are produced usually have quite an unpredictable course. *E. coli* is an example of a multidrug-resistant and ESBL-producing bacterium that can be the source of extremely severe infections [72,73,74]. As has previously been stated, some strains of *E. coli* can also cause very serious medical conditions connected with urinary and gastrointestinal tract and central nervous system [75]. On the other hand, the side effects of a prolonged usage of antibiotics include the occurrence of antibiotic resistance [76,77,78]. Today we have evidence that people can get antibiotic-resistant *E. coli* directly or indirectly from the environment [79,80]. Therefore, it is very important that we first evaluate the existence of drug-resistant *E. coli* in our surroundings and based on such findings try to outline the human and veterinary healthcare guidelines [81,82,83,84,85,86].

This paper aims to describe how people have facilitated the evolution of *E. coli’s* antibiotic resistance, while also presenting the specific mechanisms that this bacterium has developed over time to protect itself from the most typically prescribed and consumed antibiotics.

## 2. Usage of Antibiotics in Different Countries of EU Region and Spread of *E. coli* Resistance to Antibiotics

It is absolutely clear to us today that the antibiotic resistance of *E. coli* and some other bacteria involves a combination of different factors [87,88]. Research results indicate that *E. coli* exhibits the strongest resistance to the longest used and most commonly prescribed antibiotics [89,90,91]. This is exactly the case with sulfonamides, which were first used in humans around 1930s [92]. Some twenty years later, the first resistant strains of *E. coli* appeared and with time this resistance only grew stronger. It has also been found that low-income [93] and mid-income countries (Table 1) are regions with the highest antibiotic-resistance rates and it is precisely in these regions that we see the highest consumption of antibiotics [94]. On the other hand, high-income nations show a lower rate of antibiotic resistance, resulting from lower usage of antibiotics. In some high-income countries the consumption is high, for example in Belgium, France, and Italy. This is even more complex when comparing to low-income countries where on one hand the consumption may be high but the availability of many of the more advanced antimicrobials is limited [95].

**Figure 1:**
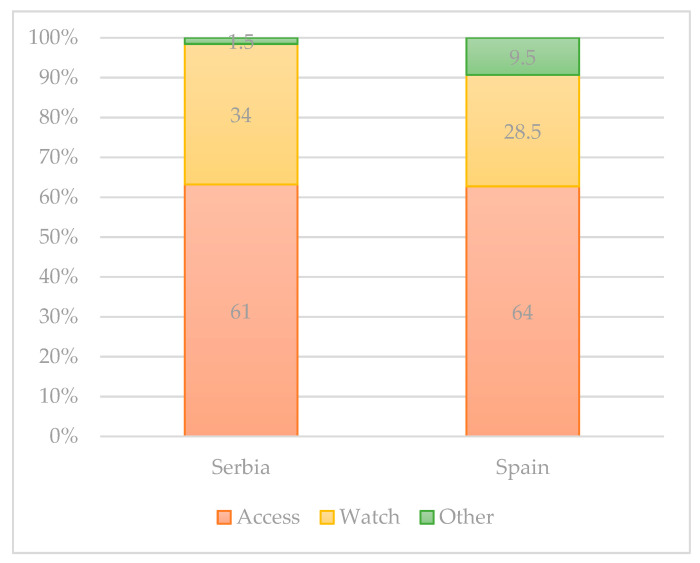
Proportional consumption of antibiotics by AWaRe categorization, % [93,96].

In the 2017 revision of the WHO Model List of Essential Medicines, antibiotics in the list were grouped into three AWaRe categories: Access, Watch, and Reserve. According to the WHO AWaRe categories [96], the classification showed that the Access group antibiotics accounted for more than 50% of total consumption both in Serbia and Spain [93]. The size of the population (in thousands) living in the European Region in 2015 was 912,984, respectively. Of the 53 Member States of the region, none is a low-income country, 20 are middle-income countries, and 33 are high-income countries. The median proportional consumption of the Access group values ranged between 61% in Spain to 64% in Serbia. The median proportion of Watch group antibiotics related to total consumption values ranging from less than 34% in Serbia and 28.5% in Spain. Reserve group antibiotics were only rarely used. The most widely used Reserve group antibiotics were intravenous fosfomycin, followed by cefepime, colistin, linezolid, and daptomycin. The antibiotics assigned to the Other group varied from 1.5% in Serbia to 9.5% in Spain (Figure 1). Overall consumption of antibiotics in these 46 countries ranged from 7.66 to 38.18 DDD per 1000 inhabitants per day. The overall absolute weight (not adjusted by population size) varied from 2.18 ton (Iceland) to 1195.69 tons (Turkey) per year.

It is a widespread opinion among scientists that antibiotic resistance has developed as the result of human activity and commonly applied treatment with antibiotics [97]. On the other hand, studies of bacteria living inside human body and other environmental bacteria helped us discover many other resistance factors that did not develop over time as a reaction to antibiotics, but were probably part of bacteria genomes in the first place [98,99,100]. Scientists often refer to those characteristics as the intrinsic resistance of bacteria [101]. It presents a great advantage of that particular bacteria strain, as its main task is to inhibit or eliminate other bacteria that live in the same environment and compete for food [102,103,104]. Hence, intrinsic resistance is different from the extrinsic antibiotic resistance, which was triggered primarily by human action [105]. In times of constantly growing antibiotic resistance and in a situation when we seem not to have any readily available antibacterial agents, it is extremely important to thoroughly study the intrinsic resistance of bacteria. That could lead to the development of a new method of fight against bacterial resistance [106]. If we could manage somehow to inhibit the factors that intrinsic resistance is composed of, perhaps bacteria would then become highly sensitive to antibiotics again. *E. coli* and other gram-negative bacteria have two important characteristics, which are the foundations of their intrinsic resistance. Namely, they have a protective impermeable membrane and a large number of efflux pumps, which successfully remove all unwanted substances from inside the cell [107,108,109].

Antibiotic resistance is an ecosystem problem threatening the interrelated human–animal–environment health under the “One Health” framework. Resistant bacteria arising in one geographical area can spread via cross-reservoir transmission to other areas worldwide either by direct exposure or through the food chain and the environment. Drivers of antibiotic resistance are complex and multisectoral particularly in lower- and middle-income countries. These include inappropriate socio-ecological behaviors; poverty; overcrowding; lack of surveillance systems; food supply chain safety issues; highly contaminated waste effluents; and loose rules and regulations. Iskandar et al. [110] have investigated the drivers of antibiotic resistance from a “One Health” perspective. They have summarized the results from many researches that have been conducted over the years and shown that the market failures are the leading cause for the negative externality of antibiotic resistance that extends in scope from the individual to the global ecosystem. Iskandar et al. [110] highlighted that the problem will continue to prevail if governments do not prioritize the “One Health” approach and if individual’s accountability is still denied in a world struggling with profound socio-economic problems.

Dsani et al. [111] investigated the spread of *E. coli* isolates from raw meat in Greater Accra region in Ghana, to antibiotics resistance, respectively. Usually, raw meat can be contaminated with antibiotic resistant pathogens and consumption of meat contaminated with antibiotic resistant *E. coli* is associated with grave health care consequences. In their research, *E. coli* was detected in half of raw meat samples. Isolates were resistant to ampicillin (57%), tetracycline (45%), sulfamethoxazole-trimethoprim (21%), and cefuroxime (17%). Multidrug resistance (MDR) was identified in 22% of the isolates. The *bla_TEM gene_* was detected in 4% of *E. coli* isolates [111]. Dsani et al. [111] concluded that levels of microbial contamination of raw meat in their research were unacceptable and highlighted that meat handlers and consumers are at risk of foodborne infections from *E. coli* including ESBL producing *E. coli*, which is resistant to nearly all antibiotics in use.

According to Hassan et al. [112], a last resort antibiotic is colistin. Colistin is crucial for managing infections with carbapenem-resistant *Enterobacteriaceae*. The recent emergence of mobile-colistin-resistance (*mcr*) genes has jeopardized the efficiency of this antibiotic. Aquaculture is a foremost contributor to the evolution and dissemination of *mcr*. Nevertheless, data on *mcr* in aquaculture are narrow. In Lebanon, a country with developed antimicrobial stewardship the occurrence of *mcr-1* was evaluated in fish. Mobile-colistin-resistance-*1* was detected in 5 *E. coli* isolated from fish intestines. The isolates were classified as multidrug-resistant and their colistin minimum inhibitory concentration ranged between 16 and 32 μg/mL. Whole genome sequencing analysis showed that *mcr-1* was carried on transmissible IncX4 plasmids and that the isolates harbored more than 14 antibiotic resistance genes. The isolates belonged to ST48 and ST101, which have been associated with *mcr* and can occur in humans and fish and help in spreading of antibiotic resistance of *E. coli*.

While, Montealegre et al. [113] have showed how high genomic diversity and heterogeneous origins of pathogenic and antibiotic-resistant *E. coli* in household settings represent a challenge to reducing transmission in low-income settings. Transmission of *E. coli* between hosts and with the environment is believed to happen more frequently in regions with poor sanitation. Montealegre et al. [113] performed whole-genome comparative analyses on 60 *E. coli* isolates from soils and fecal from cattle, chickens, and humans, in households in rural Bangladesh. Results suggest that in rural Bangladesh, a high level of *E. coli* in soil is possible led by contributions from multiple and diverse *E. coli* sources (human and animal) that share an accessory gene pool relatively unique to previously published *E. coli* genomes. Thus, interventions to reduce environmental pathogen or antimicrobial resistance transmission should adopt integrated “One Health” approaches that consider heterogeneous origins and high diversity to improve effectiveness and reduce prevalence and transmission [113].

It has been confirmed that wastewater treatment plant effluents are influenced by hospital wastewaters [114] in Germany. Alexander et al. [114] quantified the abundances of antibiotic resistance genes and facultative pathogenic bacteria as well as one mobile genetic element in genomic DNA via qPCR from 23 different wastewater treatment plant effluents in Germany. Total of 12 clinically relevant antibiotic resistance genes were categorized into frequently, intermediately, and rarely occurring genetic parameters of communal wastewaters. Taxonomic PCR quantifications of 5 facultative pathogenic bacteria targeting *E. coli*, *P. aeruginosa*, *K. pneumoniae*, *A. baumannii*, and enterococci were performed.

Since communal wastewater treatment plants are the direct link to the aquatic environment, wastewater treatment plants should be monitored according to their antibiotic resistance genes and facultative pathogenic bacteria abundances and discharges to decide about the need of advanced treatment options. Critical threshold volumes of hospital wastewaters should be defined to discuss the effect of a decentralized wastewater treatment, because they can serve as an excellent reservoir in spreading of *E. coli* resistance to antibiotic.

## 3. Inappropriate Prescribing of Antibiotics

According to scientific literature, we are now witnessing a rapid evolution of bacteria and a tremendous increase in multidrug-resistant strains largely due to selective pressure and a long-term interaction between the applied antibiotics and bacteria [115,116,117]. It seems that antibiotics have been prescribed too often and many times perhaps even inappropriately. When a person has bacterial infection and has been prescribed antibiotic treatment, what normally happens is that all susceptible bacteria get killed. However, together with the pathogenic microorganisms that caused the infection, many other microorganisms found in that specific environment will get eliminated too. On the other hand, if there are some resistant microorganisms in that environment, whether they are pathogenic or not, they will be the ones who will survive, quickly spread and outnumber all others [98,105,107].

We are all aware of the fact that millions of lives have been saved thanks to the discovery of antibiotics [118]. No wonder that this revolutionary medicine has often been considered as the “miracle drug” [118]. Unfortunately, antibiotics have been prescribed too often and sometimes even when it was not absolutely necessary [119]. Nowadays, we have a global problem, which presents an enormous threat to healthcare systems around the world. What is even more alarming is that in many countries there has not been an adequate response to this crisis. The abuse of antibiotics is still a major issue. According to the global antibiotic sales database, when we compare antibiotic consumption for the years 2000 and 2015, we can see an evident increase from around 11 doses per 1000 inhabitants per day to almost 16, which is an increase of almost 40% for the period of five years [120]. Having analyzed the statistics, together with research findings, it seems that the mean value for antibiotic consumption was largely influenced by low-income and mid-income countries [121]. These countries appear to have the largest number of multidrug-resistant bacterial infections. An even bigger problem is that studies show a considerable increase in the consumption of antibiotics such as carbapenems and colistin, which should be prescribed when everything else fails [122,123]. This could perhaps explain the emergence of *E. coli* strains resistant to precisely these antibiotics. Scientists claim that in the past there were some only very rare cases of resistance of *E. coli* to carbapenems (depending on the part of the world in question), but that in the future we may see a great increase of resistance to carbapenems [124,125]. This is mainly because of the existence of the enzymes called carbapenemases, which break down carbapenems and make them ineffective [126]. These enzymes with versatile hydrolytic capacities are plasmid-encoded and easily transmitted [127].

Medicines including vaccines are a critical component in the management of both infectious diseases and noncommunicable diseases reflected in global sales of medicines likely to exceed 1.5 trillion € by the end of 2023 and currently growing at a compounded annual growth rate of 3 to 6% [128]. Medicines also play a critical role in lower- and middle-income countries, which is in accordance with previously findings of Iskandar et al. [110]. Because usually these costs are “out-of-pocket”, there can be devastating outcomes for families when some of the members turn out to be sick. These outcomes and apprehensions are aggravated by the WHO assessing that more than half of all medicines are prescribed inappropriately, with approximately half of all patients failing to take them correctly [128].

Antibiotic resistance poses a great threat to human, animal, and environmental health. Beta-Lactam antibiotics have been successful in combating bacterial infections. Still, the overuse, inappropriate prescribing, unavailability of new antibiotics, and regulation barriers have exacerbated bacterial resistance to these antibiotics. 1,4,7-Triazacyclononane (TACN) is a cyclic organic tridentate inhibitor with strong metal-chelating abilities that has been shown to inhibit β-lactamase enzymes and may represent an important breakthrough in the treatment of drug-resistant *E. coli* bacterial strains. However, its cytotoxicity in the liver is unknown [129].

Antimicrobial stewardship is a foundation of endeavors to reduce antimicrobial resistance. To determine factors potentially influencing probability of prescribing antimicrobials for pet animals, Singleton et al. [130] analyzed electronic health records for unwell dogs (*n* = 155,732 unique dogs, 281,543 consultations) and cats (*n* = 69,236 unique cats, 111,139 consultations) voluntarily contributed by 173 UK veterinary practices. Results of their pet animal study demonstrate the potential of preventive healthcare and client engagement to encourage responsible antimicrobial drug use [130].

Robbins et al. [131] investigated the antimicrobial prescribing practices in small animal emergency and critical care. According to authors antimicrobial use contributes to emergence of antimicrobial resistance [131]. They have assumed that antimicrobial prescribing behavior varies between the emergency and critical care services in a veterinary teaching hospital, so they tried to investigate antimicrobial prescribing patterns, assess adherence to stewardship principles, and to evaluate the prevalence of multidrug-resistant (MDR) bacterial isolates. Robbins et al. [131] after investigation, which showed that the most prescribed antibiotics in emergence was amoxicillin, metronidazole, and ampicillin with the most common reasons for antimicrobial prescriptions being skin disease, gastrointestinal disease, and respiratory disease. Regarding the critical care, authors have recorded most prescribed ampicillin, enrofloxacin, and metronidazole, with the most common reasons for antimicrobial prescriptions such as gastrointestinal disease, respiratory diseases, and sepsis. Robbins et al. [131] concluded that antimicrobial prescription was common with comparable patterns. However, devotion to guidelines for urinary and respiratory infections was poor.

Lehner et al. [132] conducted the study with the objective to investigate antimicrobial prescriptions by Swiss veterinarians before and after introduction of the online ASP AntibioticScout.ch in December 2016. In the methodology, authors have used a retrospective study, where the prescriptions of antimicrobials in 2016 and 2018 were compared and their appropriateness was assessed by a justification score. The results of the study revealed that percentage of dogs prescribed antimicrobials decreased significantly between 2016 and 2018, which led to a conclusion that antimicrobials were used more carefully. The study highlights the continued need for ASPs in veterinary medicine [132].

Not only the regular hospitals and veterinary clinics have a problem with inappropriate prescribing of antibiotics, but the dentist’s clinics also have the same problem. Antibiotic resistance is a global public health problem. Around 55% of dental antibiotic prescribing is deemed inappropriate [133]. Evidence to that issue can be seen from an experiment where a total of 26 dentists were recruited for the 12-week study using a pre–post design. For six weeks, dentists self-recorded their prescription of antibiotics, analgesics, and anxiolytics. After dentists were provided education and website access, they recorded their prescription for a further six weeks. Results of the experiment reveled a substantial reduction of 44.6% in the number of inappropriate indications for which antibiotics were prescribed after the intervention and a decrease of 40.5% in the total number of antibiotics. Paracetamol with codeine substantially reduced by 56.8%. For the highly prescribed antibiotics amoxicillin, phenoxymethylpenicillin, and metronidazole, there was an improvement in the accuracy of the prescriptions ranging from 0–64.7 to 74.2–100% [133].

It is especially important that such a type of experiment showed the intervention of targeted education and the prescribing tool was effective in improving dental prescribing, knowledge, and confidence of practitioners, as well as providing an effective antibiotic stewardship tool. This context-specific intervention shows substantial promise for implementation into not only in dental practice, but veterinary and other medical practices as well.

One of the main factors that contribute to the growing antibiotic-resistance is the over-prescription of antibiotics [134]. Unfortunately, research shows that in more than 70% of cases, doctors in the US prescribed the wrong antibiotics [135]. Evidently, it is both the overuse and the inappropriate choice of antibiotics that we can blame for the antibiotic resistance that bacteria have evolved over the years [136]. In many cases, the prescribed antibiotics are suitable for acute respiratory tract infections [137,138,139,140], while for example ciprofloxacin is one of the antibiotics that is prescribed too often and inappropriately, and no wonder that *E. coli* is highly resistant to it [141,142]. Another very interesting finding shows that humans and animals with diarrhea used antibiotics quite frequently before they started experiencing the mentioned symptoms [143,144,145]. This could lead us to the conclusion that perhaps the previously used antibiotics had potentially disrupted the gut microbiota and resulted in the excessive number of pathogenic organisms resistant to drugs [117].

## 4. Mechanisms of β-Lactams Resistance towards *E. coli*

The Gram-negative bacteria called *Enterobacteriaceae* is known for its amazing capacity to become resistant to many different types of antibiotics [146,147]. *Klebsiella* and *E. coli* are the bacteria that cause the largest number of infections in humans [148], and are most often mentioned when speaking of multidrug-resistant bacteria [74,127,149]. Unfortunately, we are witnesses to the fact that *E. coli* has been increasingly developing strains that are insusceptible to the most common types of antibiotics, such as β-lactams, sulfonamides, fosfomycin, etc. [70,71,88,150]. What presents an even greater concern for doctors and scientists these days is that *E. coli* reveals resistance even to carbapenems and polymyxins, which are considered by many as the last resort antibiotics [151]. 

If we analyze the molecular structure of beta-lactams, we can see that they consist of the so-called β-lactam ring, which is supposed to inhibit the synthesis of the bacterial cell wall [70]. Beta-lactam antibiotics are specially targeted at bacterial enzymes called penicillin-binding proteins (PBPs) [152]. Unfortunately for us, bacteria have developed several methods of protection against β-lactams [153]:

1. Production of β-lactamases, which render β-lactams ineffective
2. Inhibited penetration of antibiotics to the intended location
3. Modification of the target site PBPs
4. Activation of efflux pumps

In more concrete terms, *E. coli* produces enzymes that are called “beta-lactamases” [154]. They are quite old compounds with over 2800 unique proteins [155]. The classification of β-lactamases is based on their function and structure [156]. Throughout literature, the most frequently used classification of beta-lactamases is the Ambler classification [157]. It focuses on the similarity of structure and according to this classification we can divide proteins into four main groups: the classes A, C, and D of serine-β-lactamases and the class B of metallo-β-lactamases [157]. 

Gram-negative bacteria are capable of producing different β-lactamases [156]. From the scientific point of view, the most important beta-lactamases that *E. coli* produces are carbapenemases [158], the extended-spectrum beta-lactamases (ESBL) [159], and AmpC beta-lactamases (AmpC) [160].

### 4.1. Prevalence of Antibiotic Resistance in E. coli Isolates by Disk Diffusion Method

**Figure 2:**
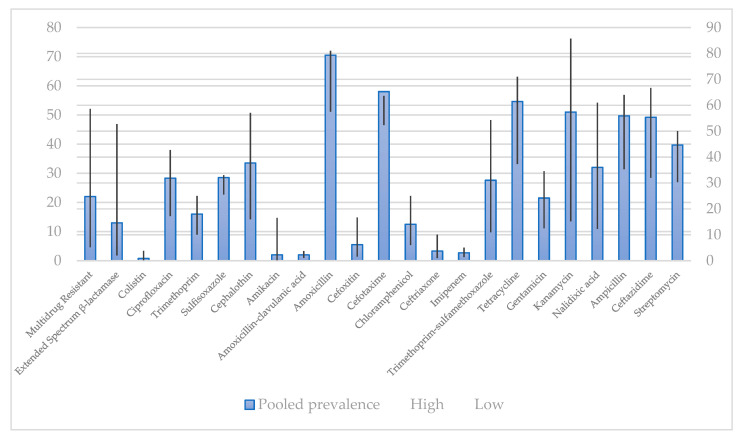
Pooled prevalence of antibiotic resistance isolates in humans by disk diffusion method, % [161].

**Figure 3:**
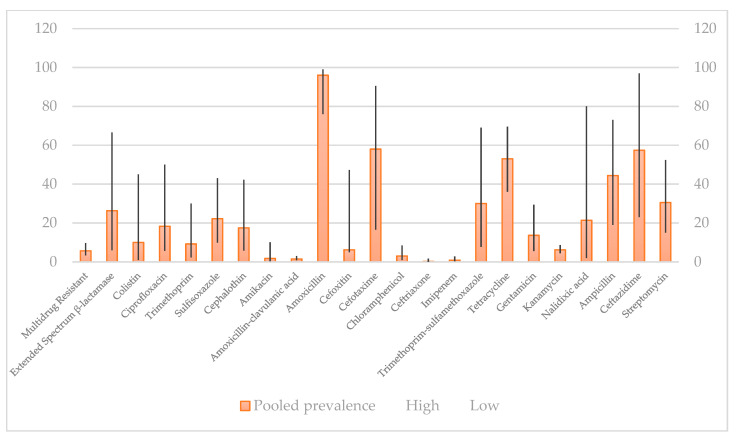
Pooled prevalence of antibiotic resistance isolates in pet animals by disk diffusion method, % [161].

Prevalence of selected antibiotic resistance in *E. coli* strains isolated from humans and pet animals is shown in Figure 2 and Figure 3.

As shown in Figure 2, highest rate of resistance of *E. coli* to amoxicillin were observed while the lowest rate of resistance was observed in colistin.

The same case as in Figure 2 was shown in Figure 3 regarding the resistance of *E. coli* to amoxicillin in pet animals, while the lowest rate of resistance was observed in ceftriaxone, respectively. This analysis nicely illustrates the evolution of antibiotic resistance and can be used for describing drug-resistance prevalence in the most recent *E. coli* strains. What is more, it shows a significantly higher prevalence of extended-spectrum beta-lactamase in pet animal isolates than in human isolates.

### 4.2. Prevalence of Antibiotic Resistance in E. coli Isolates by Minimum Inhibitory Concentration

**Figure 4:**
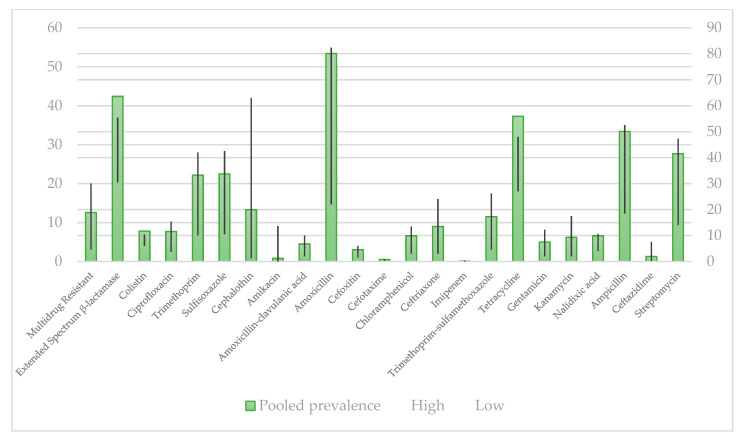
Pooled prevalence of antibiotic resistance isolates in humans by minimum inhibitory concentration, % [161].

**Figure 5:**
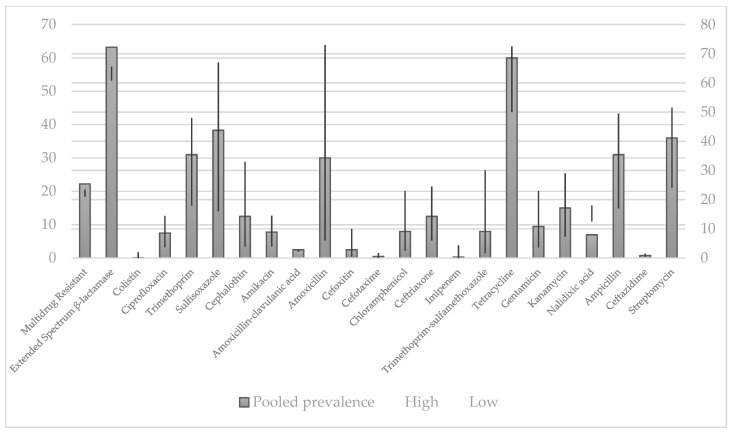
Pooled prevalence of antibiotic resistance isolates in pet animals by minimum inhibitory concentration, % [161].

Prevalence of selected antibiotic resistance in *E. coli* strains isolated from humans and pet animals by minimum inhibitory concentration (MIC) method is shown in Figure 4 and Figure 5.

As shown in Figure 4, in *E. coli* strains obtained from humans, the bacterium showed the lowest resistance to imipenem, while it exhibited the highest resistance to amoxicillin. These data are not completely in accordance with the data shown in Figure 2, where the lowest rate of resistance was observed in colistin, compared to those ones recorded for imipenem, respectively [161].

When pet animal isolates were analyzed, the lowest resistance rate was found for colistin, while the highest resistance was exhibited to tetracycline. Compared to isolates in pet animals by disk diffusion method (Figure 3), the lowest resistance identified was to ceftriaxone, while the highest resistance was found to amoxicillin, which is in accordance with data showed in Figure 5 obtained by the MIC method.

## 5. Conclusions

*E. coli* colonizes human and animals’ gut, which facilitates its spreading from fecal matter to the mouth. Due to its fascinating capacity to transfer drug resistance to other microorganisms and also acquire it from others that share the same environment, we can speak of *E. coli’s* huge evolutionary advantage. The antibiotic resistance genes are located on plasmids, which enables the easy horizontal spread of antibiotic resistance among different bacteria and, thus, poses a serious threat to medicine. With time, *E. coli* has developed several methods for neutralizing the power of antibiotics. Unfortunately, only one strain of *E. coli* can possess resistance genes that can fight off several different types of antibiotics, which makes the whole situation even more complicated for patients with bacterial infections. 

The phenomenon of antibiotic resistance in bacteria is multifactorial and depends on an interplay of a number of factors, but the common denominator is clearly the overuse of antibiotics, both in humans and animals. Therefore, the whole world is seriously in need of antibiotic or antimicrobial stewardship programs, which are supposed to prevent the overuse of antibiotics and, thus, reduce antibiotic resistance. On the other hand, all that is not enough if some socioeconomic issues remain unresolved, such as poor hygiene, lack of drinking water, or bad living conditions and overcrowded households. These factors only add to the severity of the problem of antibiotic resistance and go beyond simply restricting the consumption of antibiotics. Obviously, knowing all of the above-mentioned facts, the solution to the problem of antibiotic or multidrug resistance is not a simple one, but requires integrated efforts on all sides. It is of vital importance to closely and continuously monitor hygiene conditions in hospitals as well as waste disposal methods. As far as the treatment of patients with bacterial infections is concerned, such patients need to be carefully examined in order to bring the right decision regarding the choice of antibiotic to be given. We need to continue evaluating antibiotic-sensitivity in humans and animals while also working on the development and implementation of reliable antibiotic strategies. 

If we tackle this issue seriously and responsibly and undertake all the necessary corrective actions, we may regain control over *E. coli* infections, both in Europe and the whole world.

## Figures and Tables

**Table 1:** The consumption of total antibiotics in Defined Daily Doses, in DDD per 1000 inhabitants per day in countries of European region based on WHO database [93].

Country	DDD/1000 Inhabitants Per Day	Country	DDD/1000 Inhabitants Per Day
Albania	16.41	Kosovo	20.18
Armenia	10.31	Kyrgyzstan	17.94
Austria	12.17	Latvia	13.30
Azerbaijan	7.66	Lithuania	15.83
Belarus	17.48	Luxemburg	22.31
Belgium	25.57	Malta	21.88
Bosnia and Herzegovina	17.85	Montenegro	29.33
Bulgaria	20.25	Netherlands	9.78
Croatia	20.28	Norway	16.97
Cyprus	27.14	Poland	24.30
Czech Republic	17.18	Portugal	17.72
Denmark	17.84	North Macedonia	13.42
Estonia	12.13	Romania	28.50
Finland	18.52	Russia	14.82
France	25.92	Serbia	31.57
Georgia	24.44	Slovakia	24.34
Germany	11.49	Slovenia	13.48
Greece	33.85	Spain	17.96
Hungary	16.31	Sweden	13.73
Iceland	17.87	Tajikistan	21.95
Ireland	23.27	Turkey	38.18
Italy	26.62	United Kingdom	20.47
Kazakhstan	17.89	Uzbekistan	8.56

## Data Availability

Data is contained within the article.
